# Experimental and theoretical investigations of four amine derivatives as effective corrosion inhibitors for mild steel in HCl medium†

**DOI:** 10.1039/d0ra03560b

**Published:** 2020-06-24

**Authors:** Yasmine Boughoues, Messaoud Benamira, Lyamine Messaadia, Nafila Bouider, Samia Abdelaziz

**Affiliations:** Laboratory of Interaction Materials and Environment (LIME), University of Mohamed Seddik Benyahia B.P. 98, Ouled Aissa 18000 Jijel Algeria m_benamira@univ-jijel.dz benamira18@yahoo.fr; Department of Chemistry, University of Mohamed Seddik Benyahia B.P. 98, Ouled Aissa 18000 Jijel Algeria; Laboratoire Énergétique Appliquée et Matériaux, Université de Jijel B.P. 98, Ouled Aissa 18000 Jijel Algeria

## Abstract

Four amine derivative compounds were synthesized: 2-[(phenylamino)methyl]phenol, 2-{[(4-hydroxyphenyl)amino]methyl}phenol, 2-[(2-hydroxybenzyl)amino]benzonitrile and 2-{[(3-chlorophenyl)amino]methyl}phenol. The structure of the organic molecules was confirmed by FT-IR, ^13^C NMR and ^1^H NMR spectroscopy analyses. Their corrosion inhibition performances on mild steel in 1 M HCl were investigated using electrochemical measurements and surface analysis. Scanning electron microscopy analysis confirms the presence on the mild steel surface of a protective film of the as-prepared organic compounds, which depends on the substituent groups. Moreover, density functional theory and molecular dynamics simulation were employed in order to determine the adsorption mechanism and the position of amine derivative molecules towards the mild steel surface in an aggressive solution and to confirm the electrochemical results. The inhibition efficiency (IE) decreases with a decrease in concentration and the adsorption obeyed the Langmuir isotherm. The substitution of the OH group on the aromatic ring by Cl or CN increases IE to 90.23 and 92.56%, respectively. Molecular dynamics simulations attested that the four molecules were adsorbed on the Fe (110) surface in a flat position in the presence of water and HCl with high interaction between the different groups of the inhibitors and mild steel surface.

## Abstract 2

Four amine derivative compounds were synthesized and used as organic corrosion inhibitors.

## Graphical abstract

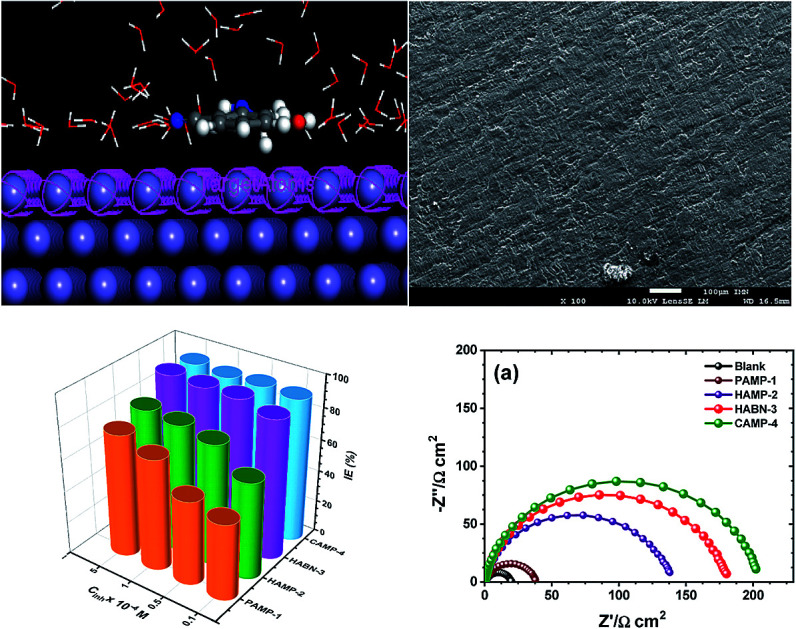

## 1. Introduction

Nowadays, steel is used in many industrial applications, such as the gas and oil processing industry, refining and extraction, water pipes, cooling water systems, boilers, and skyscrapers. Its wide use as an industrial input is helped by its low cost, availability, and ease of manufacture. However, steel remains sensitive to different forms of corrosion. The latter is defined as a physicochemical interaction between metal and the environment, leading to the change in metal properties, and also to degradation. Indeed, corrosion is a harmful phenomenon that reduces material properties and renders them unusable.

In this context, using organic inhibitors is a practical alternative to protect metals against dissolution in corrosive environment. The efficiency of organic inhibitors depends largely on the nature of the medium, metal surface state and the structure of the inhibitor. Today, it is admitted that polar functional groups containing heteroatoms such NO_2_, NH_2_, OH, CN, Cl and COOH are considered as active centers for adsorption processes between metals and an inhibitor.^1–5^ Also, it is agreed that the presence of π-electrons, aromatic rings and non-bonding electrons in the structure can facilitate the transfer of electrons to a metallic surface and cover uniformly a large surface that could increase the inhibitor efficiency.^4–7^ Meanwhile, adsorption mechanism of organic molecules involves two types of interaction: physical and chemical. The physical adsorption is associated with the charged molecules and metal surface electrostatic interaction. In contrast, the chemisorption process involves charge sharing between the heteroatoms (N, O, S, P, *etc.*) and/or an aromatic ring in the organic inhibitor and the metal surface (d-orbital). The adsorption of molecules may also occur by the association of the two interaction mechanisms.^6–8^

Over the past decade, modeling methods based on the quantum chemistry have proved their effectiveness in solving the problems of applied chemistry. For this reason, experimenters have increasingly resorted to theoretical studies by calculating the quantum chemical parameters to explain corrosion inhibition processes by organic molecules. Density Functional Theory (DFT) and molecular dynamics simulations (MD) represent powerful techniques that are used to explore inhibition performance and mechanism.^7–11^

The main objective of this study is the investigation of the corrosion inhibition of some amine derivatives in the HCl solution for mild steel. The amine derivatives have been extensively used as organic inhibitors and, in particular, in acid medium and in oil industry.^12–15^ Our amine derivatives inhibitors are characterized through ^13^C and ^1^H NMR spectroscopy analyses. Furthermore, the effect of the functional groups OH, CN and Cl on the corrosion inhibition is experimentally examined by the potentiodynamic polarization and electrochemical impedance spectroscopy (EIS) techniques as well as the surface analysis (Scanning electron microscopy, SEM). Moreover, the quantum chemical parameters such as energies of LUMO (*E*_LUMO_) and HOMO (*E*_HOMO_), energy gap (Δ*E*), a number of transferred electrons (Δ*N*) and dipole moment (*μ*) have been calculated by DFT. Finally, this study is completed by MD simulation in order to establish the orientation and the adsorption position of the amine derivative molecules at the interface Fe (110)/electrolyte. It is well-known that the flat orientation of corrosion inhibitors covers a large surface of metal which is considered as a better configuration, compared to the non-planer orientation.^16–20^

## 2. Materials and methods

### 2.1 Preparation of mild steel sample and solution

The samples of mild steel were connected to Cu-wires by conductive silver lacquer. After drying in ambient air, the mild steel sample with an exposed surface of 1–1.5 cm^2^ was mounted in epoxy resin. The elemental composition of the mild steel used in this study is checked by JEOL JSM-5800LV microscope using the energy-dispersive X-ray (EDX) spectrometer. The mild steel samples had the chemical composition in wt% of C 0.38%, P 0.09%, Si 0.31%, Al 0.01%, S 0.05%, Mn 0.05%, and Fe balance. Before each test, the sample surface was mechanically abraded with SiC papers of 600, 800 and 1200 grades, then it was cleaned with ethanol and distilled water in ultrasonic bath, and dried in air. The aggressive acid solutions of HCl (1 M) were prepared by dilution of analytical grade (37% HCl).

### 2.2 Electrochemical measurements and surface morphology

Corrosion behavior of the mild steel in HCl (1 M) solution with the presence of organic inhibitors was analyzed by potentiodynamic polarization and EIS. The experiments were carried out by Solartron Analytical 1287A potentiostat and Solartron Analytical Frequency Response Analyzers 1260. The standard three-electrode cell with Pt, mild steel and saturated calomel electrodes was used, respectively. The EIS measurements were recorded over a frequency range (10^5^ to 10^−2^ Hz) with signal amplitude of 10 mV at open circuit potential (OCP). SEM analysis was performed on a FEG-SEM JEOL 7600.

### 2.3 Synthesis of inhibitors and material characterization

The four amine derivatives (Table 1) were synthesized according to our previous work.^2^ The intermediate imines were synthesized from aniline, 4-aminophenol (3 mmol), 3-chloroaniline (3 mmol) or 2-aminobenzonitrile (3 mmol) and salicyl aldehyde (3 mmol) in methanol (10 mL), stirred at 60 °C for 15 min. After that, the different obtained imines with functional group were reduced by NaBH_4_ in MeOH at 0 °C for 15–60 min under magnetic stirring to obtain the amine derivatives shown in Table 1. The final organic inhibitors were obtained after filtration and recrystallization using water–methanol mixture. The molecule structure was confirmed by ^13^C and ^1^H NMR (Fig. S1 and S2†) and FT-IR spectroscopies.

**Table 1:** Name, abbreviation and chemical structure of the inhibitors

Structure	Name	Abbreviation
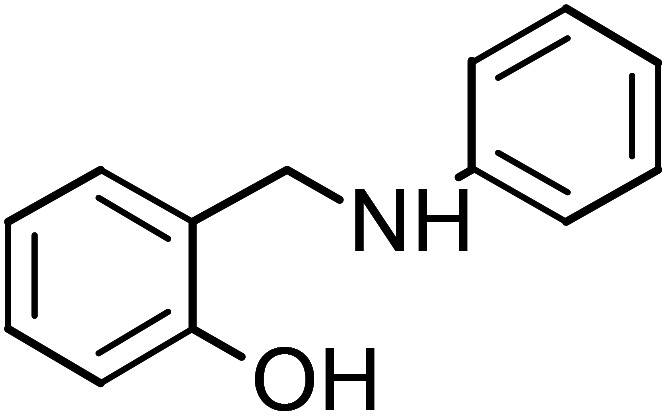	2-[(Phenylamino)methyl]phenol	PAMP-1
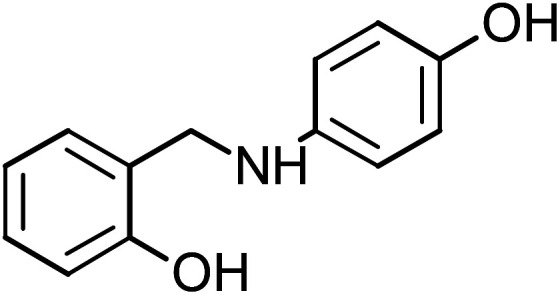	2-{[(4-Hydroxyphenyl)amino]methyl}phenol	HAMP-2
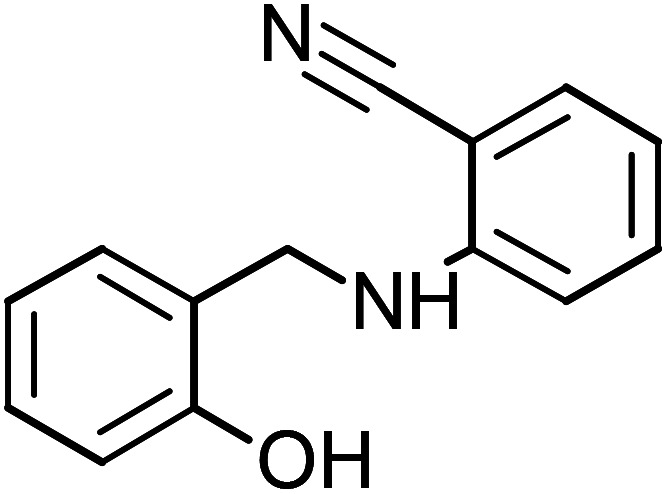	2-[(2-Hydroxybenzyl)amino]benzonitrile	HABN-3
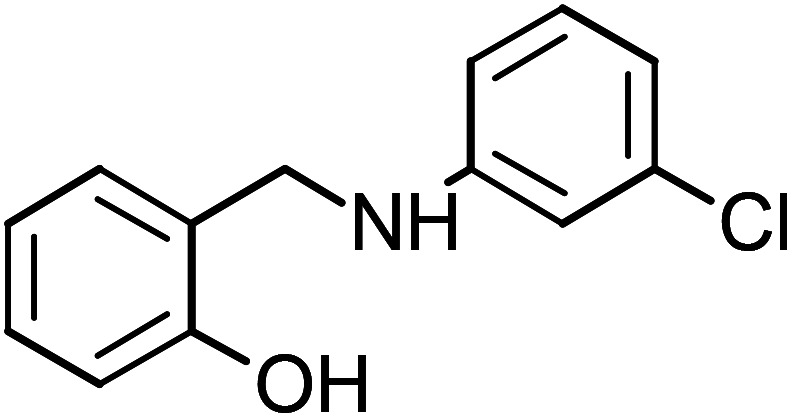	2-{[(3-Chlorophenyl)amino]methyl}phenol	CAMP-4

The FT-IR spectra were recorded on a BRUKER spectrophotometer between 400 and 4000 cm^−1^. Bruker Avance-400 MHz spectrometer was used to measure the ^1^H and ^13^C NMR spectra. TLC (Thin-layer chromatography) analysis was carried out on pre-coated silica gel plates 60F_254_ (Merck). The physical constants of the four compounds prepared are given below.

2-[(Phenylamino)methyl]phenol (PAMP-1), mp: 122.1–123.2 °C; IR (cm^−1^) *ν*: 3200 (N–H), 1560–1640 (N–H str.), 1650–2000 (C–H str.), ^1^H-NMR (CD_3_OD) *δ* (ppm): 2.29 (d, 1H, *J* = 1.7–3.5 Hz), 3.32 (d, 2H, CH_2_), 4.19 (s, 1H, NH), 5.89 (d, 1H, Ar-H, *J* = 5.6 Hz), 6.29–6.67 (m, 2H), 6.6–7.11 (m, 3H), 7.18 (m, 2H, *J* = 1.1–7.4 Hz), 9.43 (s, 1H, OH), ^13^C-NMR (CD_3_OD) *δ* (ppm): 40.045 (C_7_), 116.037 (C_1_, C_2_), 127.893 (C_3_), 129.246 (C_8_), 149.274 (C_5_), 155.686 (C_4_). Anal. cal. for C_13_H_11_NO (*M* = 197.23254 g mol^−1^): C, 79.16; H, 5.62; N, 7.10; O, 8.11%.

2-{[(4-Hydroxyphenyl)amino]methyl}phenol (HAMP-2), mp: 134–135.7 °C; IR (cm^−1^) *ν*: 3200 (N–H), 3000–3100 (C–H_Ar_), 1667–2000 (C–H str.), ^1^H-NMR (CD_3_OD) *δ* (ppm): 3.39 (d, 2H, CH_2_), 4.25 (s, 1H, NH, *J* = 106.5 Hz), 6.30 (d, 2H, Ar-H), 6.8 (d, 1H, Ar-H, *J* = 8 Hz), 6.85 (d, 1H, Ar-H), 7.04 (d, 2H, Ar-H), 9.48 (s, 1H, OH). ^13^C-NMR (CD_3_OD) *δ* (ppm): 39.937 (C_7_), 115.22 (C_1_, C_2_), 119.218 (C_8_), 132.944 (C_6_), 139.634 (C_9_, C_13_), 142.271 (C_10_, C_12_), 148.976 (C_5_), 155.295 (C_3_), 157.33 (C_11_), 160.635 (C_4_). Anal. cal. for C_13_H_11_NO_2_ (*M* = 213.2319 g mol^−1^): C, 73.23; H, 5.20; N, 6.57; O, 15.01%.

2-[(2-Hydroxybenzyl)amino]benzonitrile (HABN-3), mp: 120–120.7 °C; IR (cm^−1^) *ν*:3200 (N–H), 2200 (C≡N), 2300 (C–H str.), ^1^H-NMR (CD_3_OD) *δ* (ppm): 2.5 (d, 2H, CH_2_), 4.34 (s, 1H, NH), 6.52–6.59 (d, 2H, Ar-H), 6.75–6.80 (d, 1H), 6.8 (d, 1H), 7.43 (s, 1H, OH). ^13^C-NMR (CD_3_OD) *δ* (ppm): 40.046 (C_7_), 95.138 (C_9_), 111.861 (C_10_, C_11_), 116.132 (C_1_, C_2_), 118.856 (C_6_), 125.178 (C_13_), 128.286 (C_8_), 133.232 (C_5_), 134.601 (C_14_), 151.69 (C_3_), 159.372 (C_4_). Anal. cal. for C_14_H_10_N_2_O (*M* = 222.242 g mol^−1^): C, 75.66; H, 4.54; N, 12.60; O, 7.20%.

2-{[(3-Chlorophenyl)amino]methyl}phenol (CAMP-4), mp: 112–114 °C; IR (cm^−1^) *ν*: 2900 (N–H), 1560–1640 (N–H str.), 2300 (C–H str.), 1230 (C–O). ^1^H-NMR (CD_3_OD) *δ* (ppm): 2.47–2.56 (s, 2H, CH_2_), 3.39 (s, 1H, NH), 4.18 (d, 1H, Ar-H, *J* = 5.3 Hz), 6.46–6.66 (t, 1H, Ar-H), 6.83 (d, 1H, Ar-H, *J* = 8 Hz), 7.03 (s, 1H), 7.16 (d, 2H, Ar-H). ^13^C-NMR (CD_3_OD) *δ* (ppm): 39.961 (C_7_), 111.562 (C_11_), 115.547 (C_1_, C_2_), 125.597 (C_8_), 128.257 (C_3_), 130.613 (C_9_), 134.278 (C_10_), 151.03 (C_5_), 155.68 (C_4_). Anal. cal. for C_13_H_10Cl_NO (*M* = 231.6776 g mol^−1^): C, 67.39; H, 4.35; Cl, 15.30; N, 6.05; O, 6.91%.

### 2.4 Theoretical studies: DFT and MD simulation

Quantum chemical calculations were carried out using Gaussian 09 software package.^21,22^ The geometry optimization of the studied compounds was carried out with DFT method using the hybrid functional density B3LYP^23^ and the functional correlation of Lee–Yang–Parr,^24^ with 6-31G (d,p) basis set for all atoms. Stationary points of our molecules have been positively identified for minima with no imaginary frequencies (NIMAG = 0). The quantum chemical parameters, such as *E*_HOMO_, *E*_LUMO_, Δ*E*, *μ*, Δ*N*, electronegativity (*χ*), hardness (*η*), softness (*σ*) ionization potential (*I*) and electron affinity (*A*) from the amine derivatives to the mild steel atom were computed using the following relations:1
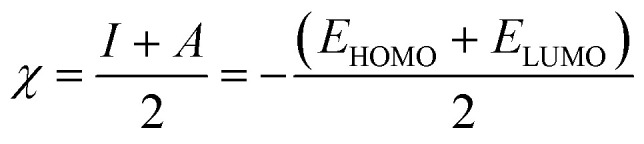

2 *I* = −*E*_HOMO_

3 *A* = −*E*_LUMO_

4
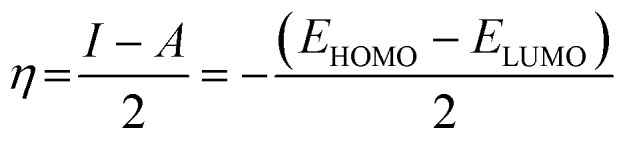
5
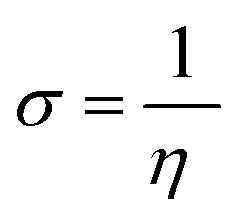
6
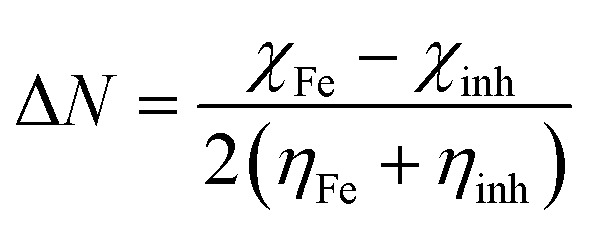


Therefore, *I* and *A* quantities are related to *E*_HOMO_ and *E*_LUMO_ with the Koopmans theorem.^25^*χ*_Fe_ (7 eV mol^−1^) and *χ*_inh_ designate electronegativity of Fe and inhibitor molecule, respectively. The theoretical values of iron hardness (*η*_Fe_) is equal to 0 eV mol^−1^,^26–28^ assuming that *I* = *A* for metallic bulk atoms.^29^

MD simulations were carried out using the adsorption locator calculation implemented in the Material Studio 7.0 software^30^ in order to study the interaction between the inhibitor and Fe-surface in the presence of water and HCl (H^+^, Cl^−^). The Fe (110) crystal surface was chosen for its stable crystalline orientation compared to other Fe surfaces.^31,32^ The COMPASS force field was used to optimize the geometry of the title compounds. The simulation was performed in a simulation box (24.82 Å × 24.82 Å × 38.10 Å) with periodic boundary conditions. In order to avoid any arbitrary boundary effects, Fe (110) surface was cleaved with thickness of 5 Å depth using periodic boundary conditions. The Fe (110) plane that contains five layers is enlarged to a (10 × 10) supercell to provide a large surface area for strong iron–inhibitor interactions. A vacuum slab with 30 Å thickness was built above the Fe (110) plane. To avoid the arbitrary boundary effects, periodic boundary conditions were used on the Fe (110) surface in a 5 Å depth. The MD simulations were done at 25 °C with a time step of 1 fs and a simulation time of 500 ps. In addition, MD simulations were made to evaluate the inhibitor adsorption energies and the iron surface in 100 water molecules and one HCl (H_3_O^+^, Cl^−^).

## 3. Results and discussion

### 3.1 Potentiodynamic polarization study

**Fig. 1:**
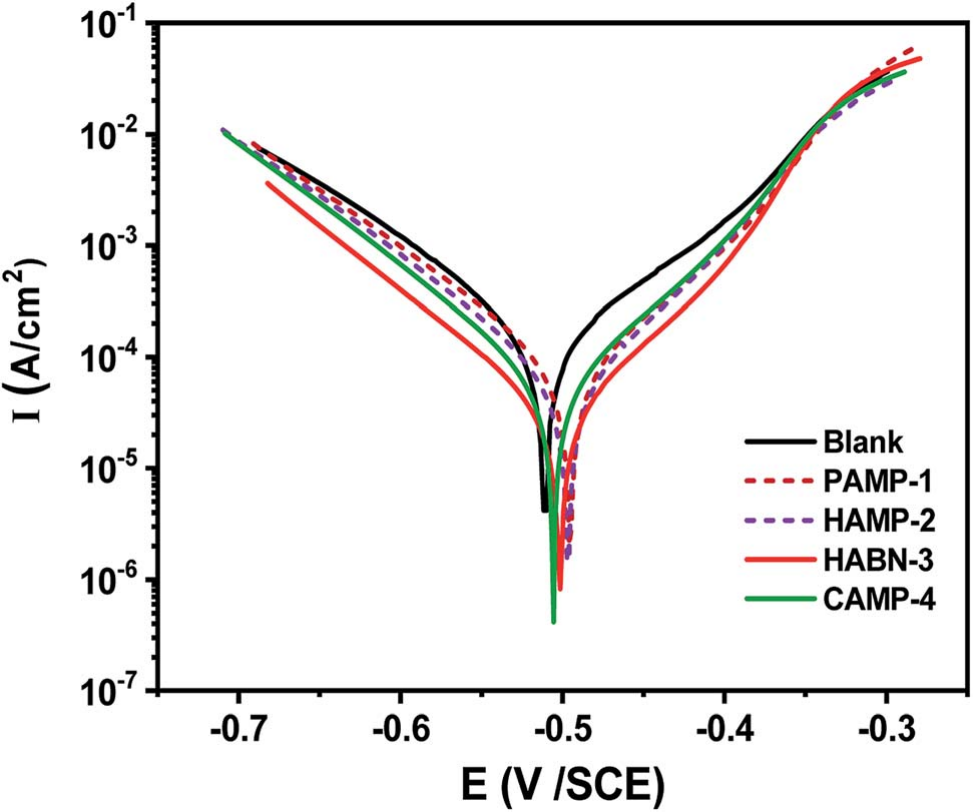
Polarization curves for mild steel in 1 M HCl containing different inhibitors of 10^−4^ M concentration at 298 K.

Potentiodynamic polarization curves shown in Fig. 1 were recorded after 2 h of immersion of mild steel in 1 M HCl with and without inhibitors to reach steady state corrosion process. The range of the applied potential is varied within *E*_corr_ − 0.25 V < *E*_corr_ < *E*_corr_ + 0.25 V under a scanning rate of 1 mV s^−1^. The corrosion current densities (*I*_corr_) and corrosion potentials (*E*_corr_) were determined by fitting the plots using the CView® software. According to the Fig. 1, the fitting parameters, such as *I*_corr_ and *E*_corr_ were listed in Table 2. The polarization resistance (*R*_p_) was determined from the linear current potential (*I*–*E*) plot in the range (*E*_corr_ ± 20 mV). The mild steel without inhibitor presents a corrosion current (*I*_corr_) of 144 μA cm^−2^ and *E*_corr_ of −0.506 V_SCE_. Our amines derivative inhibitors can be classified as a mixed-type inhibitor due to the shift of cathodic and anodic branches of Tafel lines to the lower current densities compared with the sample without inhibitor. The addition of the four inhibitors shift the corrosion potential to positive value. The displacement of *E*_corr_ is less than 33 mV and according to the literature our inhibitors can be considered as mixed type.^2,33–35^ Therefore, the mixed-type inhibitor behavior confirms the previous studies in the acidic medium of organic compounds.^2,14^ It is obvious from Tafel polarization curves that the cathodic Tafel lines remain almost parallel and unchanged which suggested that the presence of organic inhibitors do not affect the reduction of H^+^ ions. It can be seen from the anodic branch of Fig. 1 that the oxidation of the mild steel decreases gradually with the presence of inhibitors at low anodic over potentials part, below ∼−0.35 V/SCE. After that, an increase of the corrosion rate is observed for more anodic potentials. The presence of inhibitors decreases noticeably the current densities to attain 10.71 μA cm^−2^ with HABN-3 inhibitor. In this study, PAMP-1 was considered as reference compound with one functional OH group, its inhibition efficiency (IE%) was 70.64%. The addition of a second functional OH group at the second aromatic ring (HAMP-2) increases IE to reach 80.47%. The substitution of OH function by Cl (CAMP-4) or CN (HABN-3) shifts *E*_corr_ toward the anodic direction and also increases IE up to 90.23 and 92.56% for CAMP-4 and HABN-3, respectively. These results confirm that the presence of either aromatic rings (π-electrons of the double bond) or OH, CN, Cl groups on the aromatic ring is favorable to increase IE in the HCl medium.

**Table 2:** Tafel parameters after immersion in 1 M HCl solution of mild steel without and with inhibitors

	*C*_inh_ (×10^−4^ M)	*E*_corr_/SCE (V)	*I*_corr_ (μA cm^−2^)	*R*_p_ (Ω cm^2^)	IE (%)	*θ*	*V*_corr_ (μm per year)
Blank	0	−0.506	143.93	31.18	—	—	788.91
PAMP-1	0.1	−0.493	73.90	61.43	48.65	0.49	709.72
	0.5	−0.500	67.68	75.52	52.98	0.53	541.66
	1	−0.495	42.26	162.8	70.64	0.71	338.16
	5	−0.503	33.57	157.05	76.68	0.77	293.09
HAMP-2	0.1	−0.483	55.48	85.13	61.45	0.61	447.10
	0.5	−0.498	34.26	151.57	76.20	0.76	276.10
	1	−0.496	28.11	200.40	80.47	0.80	245.41
	5	−0.489	25.78	203.95	82.09	0.82	225.06
HABN-3	0.1	−0.492	17.85	300.87	87.60	0.88	121.28
	0.5	−0.496	11.64	430.40	91.91	0.92	101.61
	1	−0.500	10.71	508.04	92.56	0.93	86.34
	5	−0.473	8.04	590.30	94.41	0.94	64.78
CAMP-4	0.1	−0.502	15.58	309.82	89.17	0.89	136.04
	0.5	−0.500	14.35	350.58	90.03	0.90	125.24
	1	−0.505	14.06	315.24	90.23	0.90	135.05
	5	−0.500	11.00	400.88	92.36	0.92	87.99

**Fig. 2:**
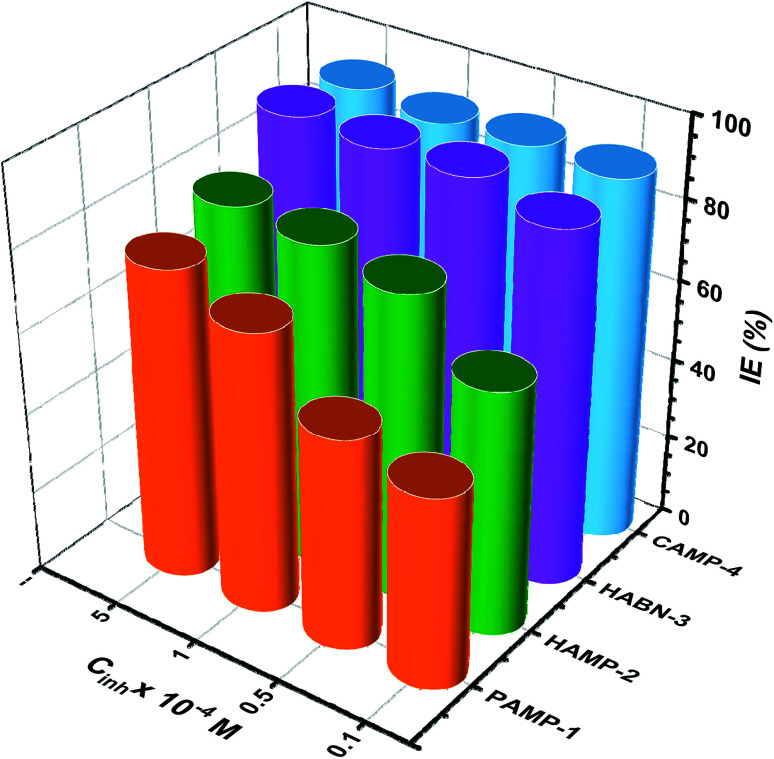
The inhibition efficiency (IE%) of inhibitors at different concentrations (10^−5^ to 5 × 10^−4^ M), *T* = 298 K.

The effect of inhibitor concentration from 10^−5^ to 5 × 10^−4^ M on IE has been studied as shown in Fig. 2. In general, it was noticed that all the inhibitors present high IE even at low concentration. The two compounds, HABN-3 and CAMP-4, confirm again their high IE which exceeds 87% regardless of the concentration. The behavior of these compounds can be attributed to the stability and the surface coverage of the protective film deposited on the metal surface even in small amounts. The inhibitor molecules were adsorbed on the mild steel/acid medium interface to form an organic film by sharing the electrons between the oxygen and nitrogen atoms and π-electron of aromatic ring and the vacant d-orbital of Fe of mild steel surface atoms. Water molecules of the metal surface were replaced by inhibitor molecules. The inhibition efficiency is largely related to the organic inhibitor structure and to the presence of different substituted groups.

### 3.2 Electrochemical impedance spectroscopy

**Fig. 3:**
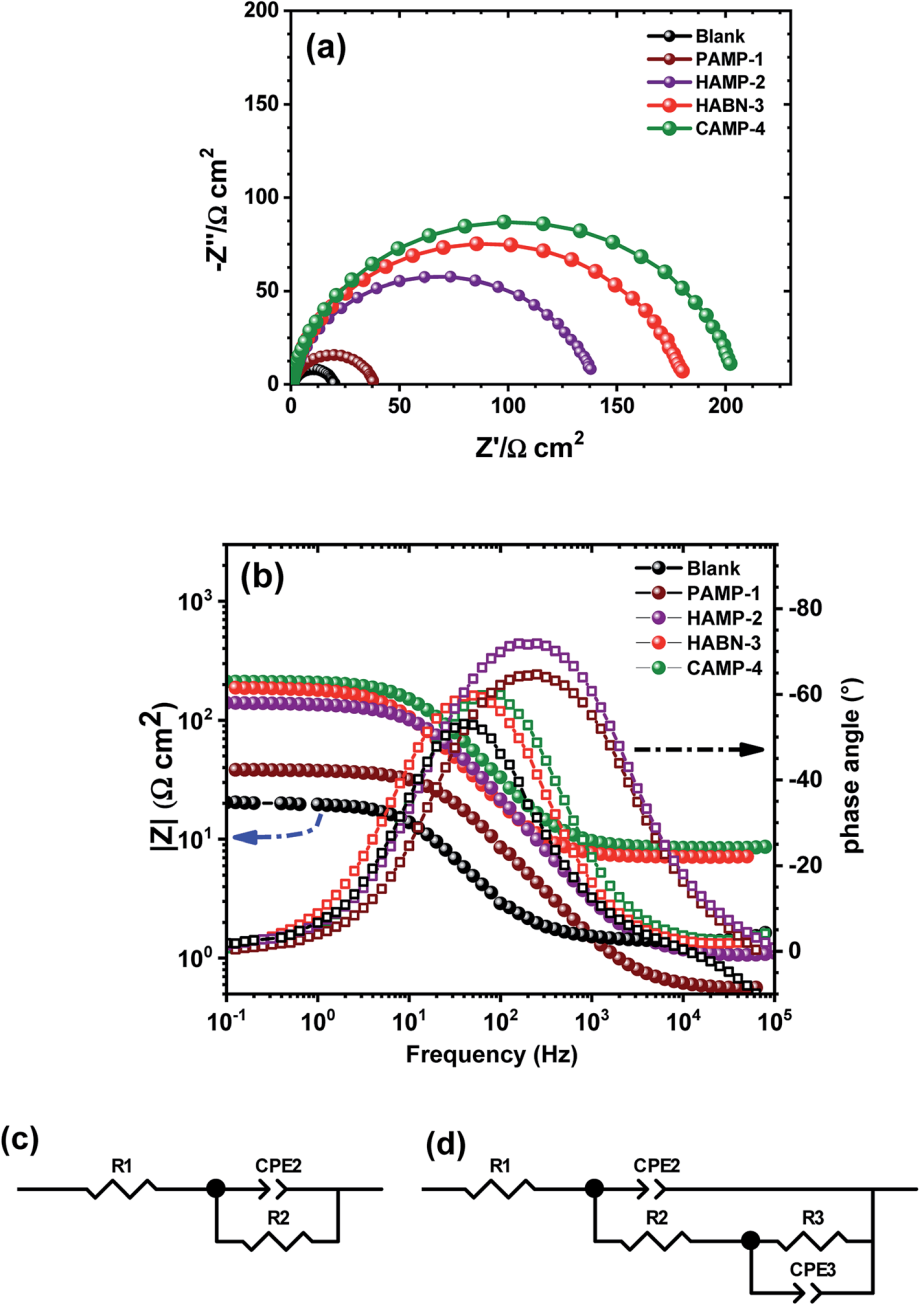
(a) Nyquist diagrams for mild steel in 1 M HCl in the absence and in the presence of the various inhibitors (10^−4^ M) at 298 K. (b) Bode diagrams. Equivalent circuits used to fit the experimental data: (c) without inhibitor (d) in the presence of inhibitors.

Electrochemical impedance spectroscopy (EIS) is well known as nondestructive powerful technique used to examine the corrosion activity at the mild steel/aggressive solution interface. EIS spectra were obtained after two hours of immersion of mild steel in 1 M HCl. As shown in the Nyquist plots (Fig. 3a), all the spectra exhibit the presence of one semicircle. The presence of inhibitors did not change the shape of the impedance spectra, which proves that the inhibitors control the corrosion process rather than its mechanism. The diameters of the semicircles of samples with inhibitors become larger than that without inhibitors, thus attesting the increase of the corrosion resistance process and the formation of a thin organic film on the surface of mild steel.

The experimental data were fitted by CView® software using the two equivalent circuit models shown in Fig. 3c and d. The first one was used to describe the behavior of the mild steel sample in HCl (1 M) without inhibitor. It consists of a series resistance (*R*_1_) attributed to the resistance of the electrolyte solution (*R*_e_), which represents the ohmic drop in the electrolytic solution, and the pseudo capacitance (CPE_2_) in parallel with the resistance *R*_2_ attributed to the transfer resistance charge (*R*_tc_), thus describing the electrical behavior at the interface electrode/solution interface. Constant Phase Element (CPE_2_) assigned to the double layer capacitance was used instead of pure capacity (*C*) due to inhomogeneity on the mild steel surface, such as the roughness as well as the formation of corrosion products or porosity.^36,37^ However, the Nyquist plots in the presence of inhibitors showed the flattening of the semicircle attesting the presence of two time constants. The phenomenon is well showed in the Bode diagram (Fig. 3b) by the presence of a more visible plateau at low and medium frequencies. These observations confirm the choice of fitting EIS diagrams by two *R*‖CPE. In this case, the second equivalent circuit model (Fig. 3d) was used to fit the experimental curves. The resistance of the aggressive electrolyte solution (*R*_1_) was placed in series with CPE_2_ assigned to the thin film formed by organic molecules and corrosion product, which is in parallel to the film resistance (*R*_2_ = *R*_f_). This resistance is placed in series with the double layer response (CPE_3_) at the interface metal/solution. This double layer capacitance is in parallel with the transfer resistance charge (*R*_3_) related to the process of corrosion.^2,38–40^ Consequently, the polarization resistance (*R*_p_) was considered as the sum of *R*_f_ and *R*_tc_.

The calculated data using the equivalent circuit and presented by lines (Fig. 3a) fit well the experimental data (symbol) with an error of the resistance (*R*) and CPE less than 3%. The EIS fitting parameters using the equivalent circuits were listed in Table 3. The addition of inhibitors strongly increased the *R*_p_ from 18.79 Ω cm^2^ to 204.46 Ω cm^2^, which is eleven times greater when CAMP-4 inhibitor was added to the sample without inhibitor. The increase of the *R*_f_ in the presence of inhibitors attests the existence and effectiveness of the organic film adsorbed on the surface of the mild steel. Such phenomenon was accompanied by the increase of the transfer resistance charge which drastically improve the corrosion resistance.

**Table 3:** EIS parameters for mild steel in 1 M HCl without and with inhibitors (10^−4^ M)

	*R*_1_ (Ω cm^2^)	*R*_2_ (Ω cm^2^)	*R*_3_ (Ω cm^2^)	*R*_p_ (Ω cm^2^)	IE (%)
Blank	1.104	18.79	—	18.79	—
PAMP-1	0.558	22.44	15.23	37.67	50.12
HAMP-2	1.074	121.80	19.20	142.00	86.77
HABN-3	0.318	134.00	47.28	181.28	89.63
CAMP-4	0.403	159.60	44.86	204.46	90.81

According to EIS results, HABN-3 and CAMP-4 inhibitors exhibit the best corrosion resistance than PAMP-1 and HAMP-2 in total agreement with potentiodynamic polarization study and confirm once again the higher inhibition activity of these compounds in HCl medium. The slight difference in IE values between polarization results and EIS may be related to the difference in the measurement principle of both techniques.

### 3.3 Adsorption isotherm

The inhibition of metal corrosion by using organic compounds can be explained by the adsorption mechanism which occurs by chemical and/or physical adsorption.

The surface coverage (*θ*) values listed in Table 1 were calculated by the relation:7
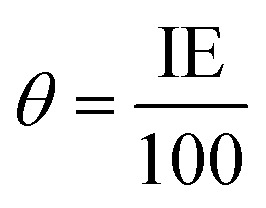


**Fig. 4:**
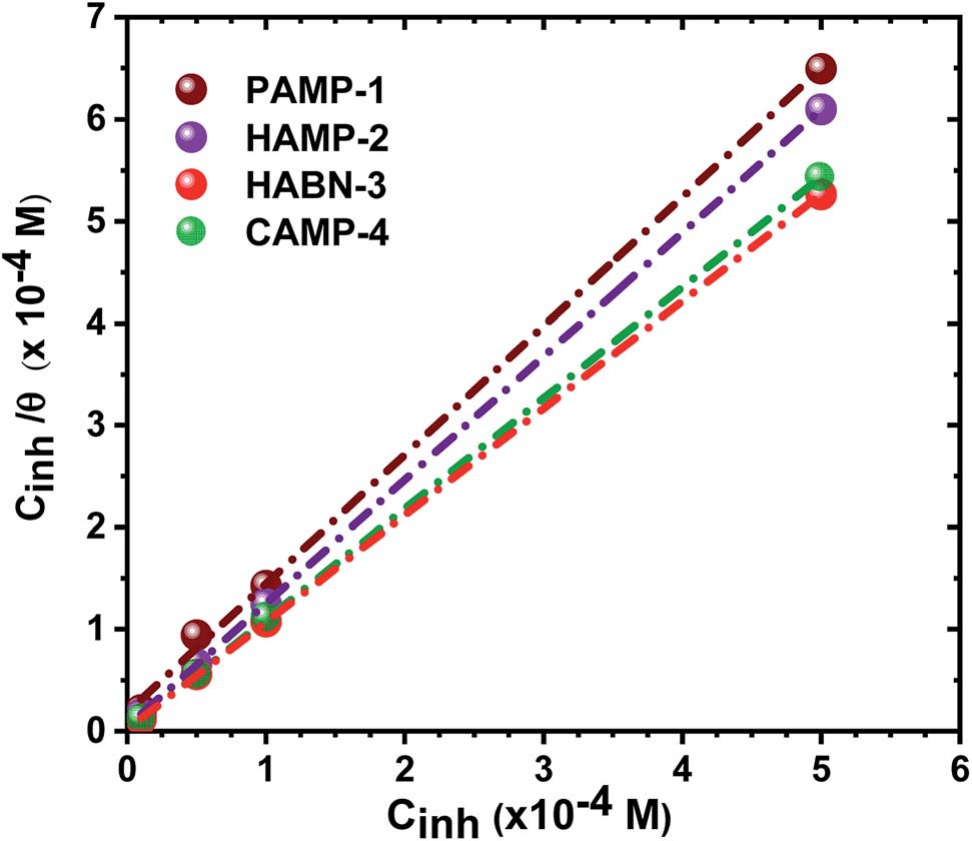
Langmuir adsorption isotherm of inhibitors in 1 M HCl for mild steel at 25 °C.

The plot of *C*_inh_/*θ versus C*_inh_ yielded straight lines with slopes and correlation coefficients (*R*^2^) closes to 1 (Fig. 4 and Table 4). The adsorption obeys the Langmuir adsorption isotherm given by the following equation:8
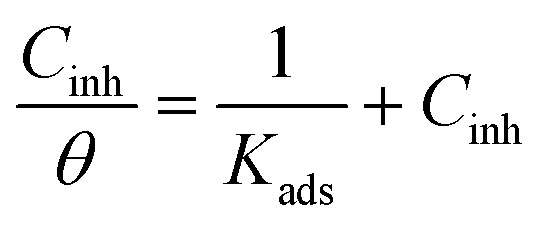
where *C*_inh_ is the inhibitor concentration and *K*_ads_ the adsorptive equilibrium constant. *K*_ads_ is related to the standard free energy of adsorption (Δ*G*^0^_ads_) according to eqn (9).9
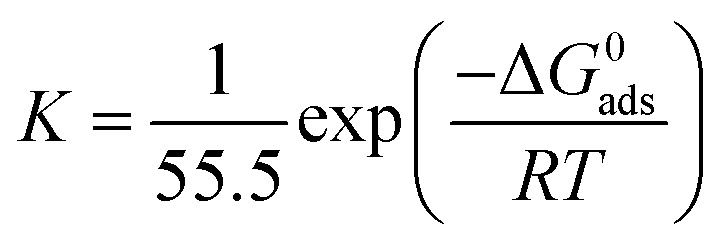
where *R* = 8.314 J K^−1^ mol^−1^, *T* (K) is the temperature and 55.5 is the molar concentration (mol L^−1^) of water.

**Table 4:** Thermodynamic parameters for the adsorption of inhibitor on mild steel in 1 M HCl at 298.15 K

	*K*_ads_ (×10^4^ L mol^−1^)	Slope	*R* ^2^	Δ*G*^0^_ads_ (kJ mol^−1^)
PAMP-1	5.43	1.26	0.984	−36.96
HAMP-2	22.22	1.21	0.999	−40.45
HABN-3	46.29	1.04	0.999	−42.27
CAMP-4	83.33	1.08	1.000	−43.73

The negative free enthalpy (Δ*G*^0^_ads_) obtained indicates a strong adsorption of the inhibitors on the surface of mild steel as well as the spontaneity of the adsorption process. In addition, the values of Δ*G*^0^_ads_ (>20 kJ mol^−1^) are a signature of electrostatic interactions between the organic compounds and the metal (physisorption), whereas Δ*G*^0^_ads_ less than 40 kJ mol^−1^, corresponds to the chemisorption due to the charge transfer between the organic molecules and the metal surface which formed coordination bonds (chemisorption).^10,41^ The Δ*G*^0^_ads_ values obtained lie between −36 and −44 kJ mol^−1^. Consequently, the adsorption of the inhibitors occurs by association of the two interaction mechanisms in the case of PAMP-1 inhibitor and mainly by chemical adsorption for the other inhibitors.

### 3.4 Surface analysis

**Fig. 5:**
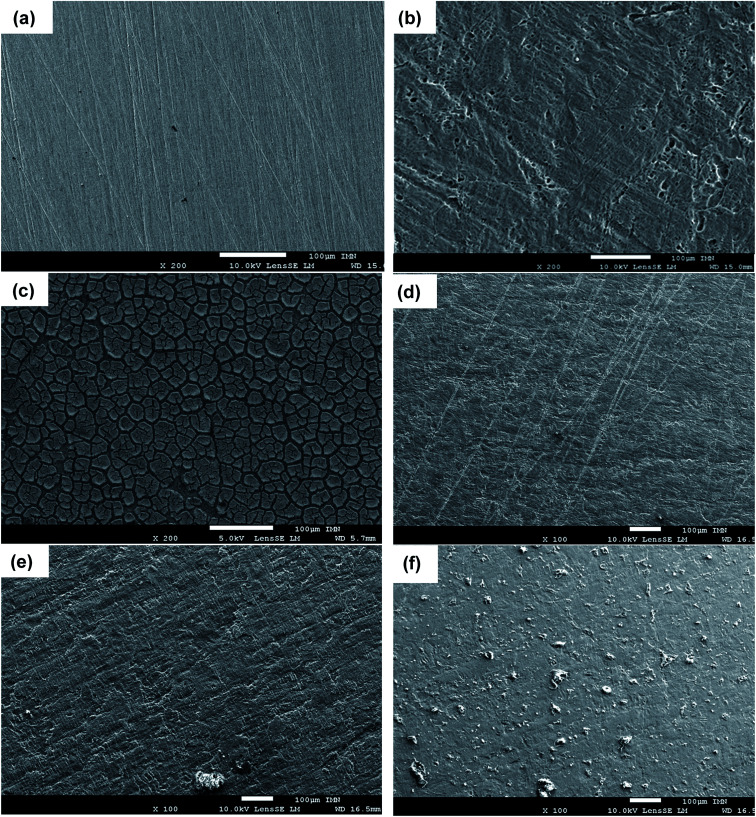
SEM images of mild steel samples before and after 24 h immersion in 1 M HCl. (a) before immersion, (b) after immersion in uninhibited solution. After immersion with 10^−4^ M of (c) PAMP-1, (d) HAMP-2, (e) HABN-3 and (f) CAMP-4 inhibitors.

To confirm the electrochemical results of the inhibitors and the formation of a protective film on the sample surface, the surface states with and without inhibitor were observed after 24 hours of immersion in the aggressive HCl solution by SEM analysis. Fig. 5a and b show the comparison between the surface micrographs of the mild steel sample before and after 24 hours of immersion in 1 M HCl. It can be shown the abrading due to the mechanical preparation of samples by SiC papers before tests (Fig. 5a). The surface is drastically damaged after immersion with the appearance of deformations at the surface of the sample (Fig. 5b). The effect of HCl was drastically limited in the presence of organic inhibitors in the solution and in particular with HAMP-2, HABN-3 and CAMP-4. Homogeneous and continuous film was deposited on the surface, thus forming a protective layer limiting the attack by reducing the anodic oxidation reaction. In the case of PAMP-1 inhibitor, the surface was not totally covered and the protective film presents some cracks and deformities which explains the low inhibition efficiency of this compound.

Indeed, the inhibition efficiency of PAMP-1 inhibitor is between 48 and 77%, and lower than that obtained for the other inhibitors. According to the earlier published studies,^2,4,36,41^ this difference in the surface state is already observed when the OH group or other groups (CN, Cl, *etc.*) are added to the structure of organic molecules and can be attributed to the beneficial effect of the presence of these groups to cover the entire surface of the mild steel as confirmed earlier by the DFT and molecular dynamics simulation used to explain such difference obtained experimentally. The results of surface analysis complete and confirm the promising performances obtained by electrochemical measurement with deposition of the synthesized compounds on the mild steel surface, which directly influences the anodic phenomena by minimizing the arrival of the aggressive electrolyte solution on the metal surface.

### 3.5 Quantum chemical study

**Fig. 6:**
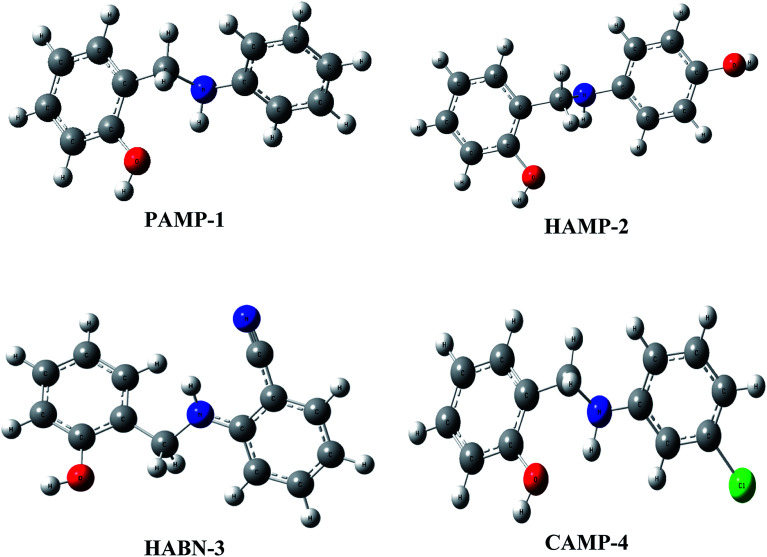
Optimized molecular structures of inhibitors.

The geometrical structures of the molecules were optimized using DFT at B3LYP/6-31G (d,p) level as shown in Fig. 6. The study is conducted to reveal the relationship between mild steel surface and organic molecules. The quantum chemical calculations present powerful tools to study the organic inhibition molecules. The frontier molecular orbitals (FMOs) are essential in fundamental chemistry and allow us to determine the chemical reactivity of the molecules responsible for the adsorption onto the mild steel surface.^42–44^

**Fig. 7:**
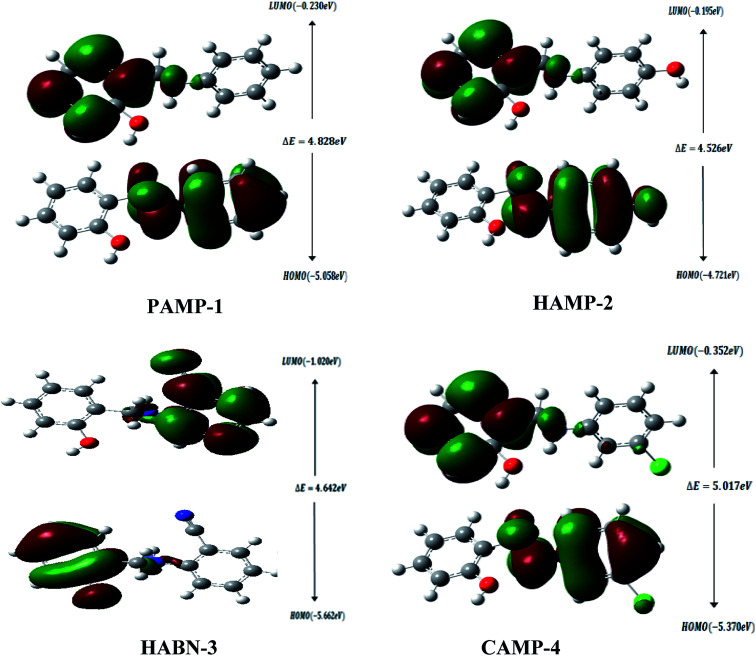
Frontier molecular orbitals HOMO and LUMO of PAMP-1, HAMP-2, HABN-3 and CAMP-4.

The high value of *E*_HOMO_ represents the ability of the inhibitor to donate electrons to the unoccupied d-orbital of the metal surface while the lowest *E*_LUMO_ indicates the ability to accept electrons from the metal surface. Fig. 7 shows the 3D plots of the frontier molecular orbitals HOMO and LUMO. Meanwhile, it is observed that the three inhibitors PAMP-1, HAMP-2 and CAMP-4 have a comparable HOMO electron density distribution. Preferential distribution is observed in the –CH_2_–NH-group and the substituted aromatic ring. This situation indicates that the ability to donate electrons is almost identical except HABN-3 molecule in which the HOMO electron density distribution is located on the –CH_2_–NH-group and phenyl ring. On the other hand, LUMO of PAMP-1, HAMP-2, and CAMP-4 molecules are also similar and the electron clouds are essentially located on the phenol ring and –CH_2_–NH-group. This attests that –CH_2_–NH-group acts as donor and acceptor center of electrons, in addition, it demonstrates that the chemical molecules adsorption can occur between the LUMO of the inhibitors and 4s^2^ orbital of Fe. Moreover, the LUMO of HABN-3 molecule is located on the substituted aromatic ring.

The results of quantum chemical calculations are summarized in Table 5. The *E*_LUMO_ values of inhibitors decrease in the following sequence, HABN-3 (−1.020 eV) < CAMP-4 (−0.352 eV) < PAMP-1 (−0.230 eV) < HAMP-2 (−0.195 eV); this suggested that the capacity to receive the electrons increases in the order, HABN-3 > CAMP-4 > PAMP-1 > HAMP-2. HABN-3 and CAMP-4 present the high affinity to accept electrons and to be adsorbed on the metal surface in perfect agreement with the Δ*G*^0^_ads_ values listed in Table 4. According to the *E*_HOMO_ values, HAMP-2 presents the highest donor capacity among the inhibitors and the highest electron transfer on the mild steel surface.

**Table 5:** Quantum chemical parameters calculated at DFT level using the 6-31G (d,p) basis set for inhibitor molecules in aqueous phase (eV)

Inhibitors	Total energy (eV)	*E*_HOMO_ (eV)	*E*_LUMO_ (eV)	Δ*E* (eV)	*μ* (D)	*I* (eV)	*A* (eV)	*χ* (eV)	*η* (eV)	*σ* (eV)	Δ*N*
PAMP-1	−17229.455	−5.058	−0.230	4.828	3.4030	5.058	0.230	2.644	2.414	0.414	0.902
HAMP-2	−19276.106	−4.721	−0.195	4.526	3.7178	4.721	0.195	2.458	2.263	0.441	1.003
HABN-3	−19739.489	−5.662	−1.020	4.642	4.7094	5.662	1.020	3.341	2.321	0.430	0.788
CAMP-4	−29735.058	−5.370	−0.352	5.017	4.7301	5.370	0.352	2.861	2.508	0.398	0.820

The energy band gap (Δ*E* = *E*_LUMO_ − *E*_HOMO_) characterizes the chemical reactivity and stability of the inhibitor molecules. The small value of Δ*E* increases and facilitates the adsorption between the molecule and metal surface through the two electron transfer ways.^45–47^ The Δ*E* values are found to increase in the following order, HAMP-2 (4.526 eV) < HABN-3 (4.642 eV) < PAMP-1 (4.828 eV) < CAMP-4 (5.017 eV). It is noted that HAMP-2 and HABN-3 possess the lowest energy band gap and partially confirm the experimental results, in particular the high inhibition of HABN-3. The values of global electronegativity *χ* suggested that HABN-3 and CAMP-4 compounds have the reducing power and highest tendency to donate electrons to the Fe surface in good accordance with the experimental inhibition efficiencies. Finally, Δ*N* is referred to the ability of inhibitor molecule to donate electrons to the vacant d-orbital of metal surface if Δ*N* > 0 or to accept electrons from the Fe surface if Δ*N* < 0. It can be seen from the Table 5 that all Δ*N* values are positive, lying between 0.820 and 1.003 eV, and attesting the electron transfer from inhibitors to Fe surface.

### 3.6 Molecular electrostatic potential (MESP)

**Fig. 8:**
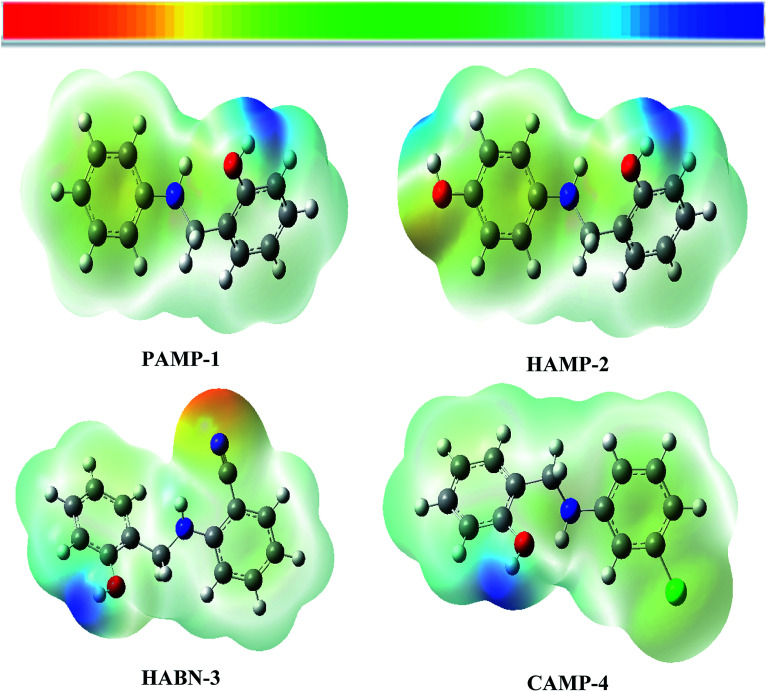
Molecular electrostatic potential surface (MESP) of inhibitor compounds PAMP-1, HAMP-2, HABN-3 and CAMP-4.

The molecular electrostatic potential (MESP) was used as an important visual technique to indicate the chemical reactivity sites. The electrostatic potential at different points on the electron density surfaces is clarified by different colors. Red color is associated with electrophilic active region, regions of most negative electrostatic potential. Blue color represents the most positive electrostatic potential regions (nucleophilic region), while the green color represents region of zero potential. The potential rises in the following order red < orange < yellow < green < blue.^48,49^ The electrostatic potential of the four compounds is shown in Fig. 8. The electron rich regions are principally localized around heteroatoms and conjugated double bonds. The oxygen, nitrogen, chloride and CN groups are indicators of negative regions which favor the electrophilic attacks. Hydrogen atoms which have blue color (positive sign) favor nucleophilic attacks.

### 3.7 Molecular dynamic (MD) simulations results

MD simulations were used to understand the corrosion inhibition mechanism of our compounds and to complete the quantum chemical calculations. Nowadays, MD is increasingly used in the field of corrosion due to encouraging results. It is considered as a powerful technique to explain the adsorption phenomena between molecule and metal surface taking into account the real conditions.^7,9,10^ DFT approach does not take into account metal–inhibitor interactions, whereas MD simulations explore all chemical entities that participate in a corrosion process.

**Fig. 9:**
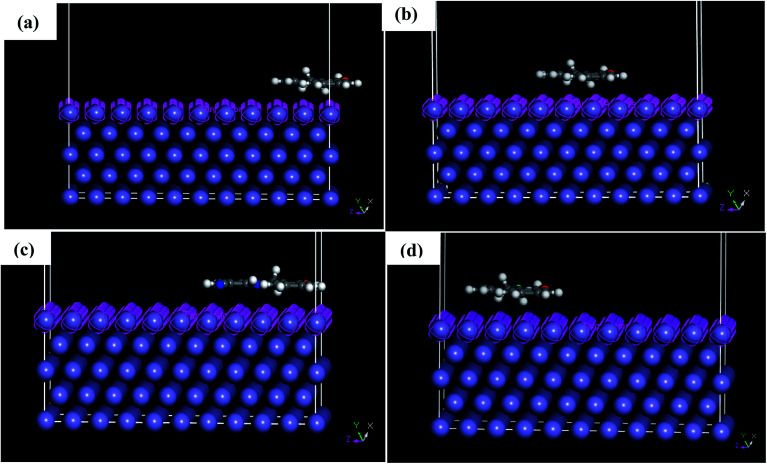
Side and top views of equilibrium configurations for the adsorption of (a) PAMP-1, (b) HAMP-2, (c) HABN-3 and (d) CAMP-4 on the Fe (110) surface.

**Fig. 10:**
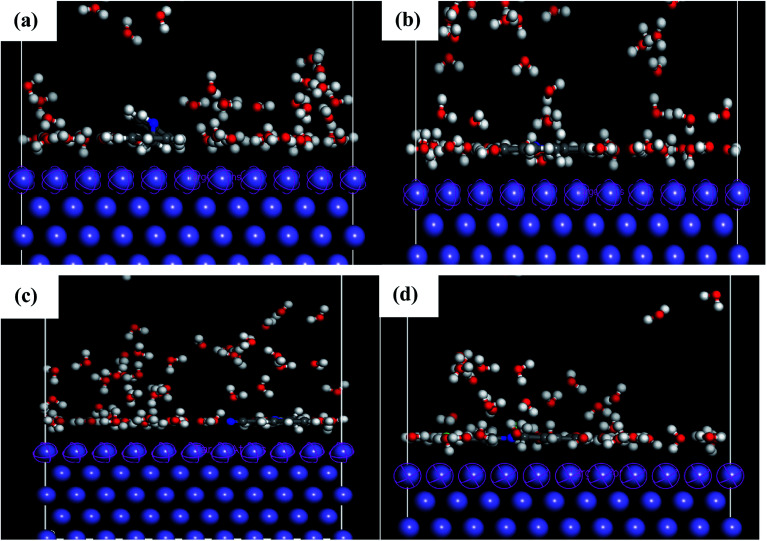
Side and top views of equilibrium configurations for the adsorption of (a) PAMP-1, (b) HAMP-2, (c) HABN-3 and (d) CAMP-4 obtained by molecular dynamic simulations on Fe (110)/100H_2_O/HCl surface.

The adsorption behavior of the inhibitors on the mild steel surface under vacuum and in the presence of 100 water molecules was studied using the molecular dynamics simulations. It can be seen from Fig. 9 that the four molecules were adsorbed on Fe (110) surface in flat position; they are completely parallel with maximum interaction between the different groups of the organic inhibitors and the metal surface. These positions are ideal to cover entirely the mild steel surface. The energies of adsorption, rigid adsorption, deformation and differential (d*E*_ads_/d*N*_i_) were calculated by molecular dynamics simulation^47,50^ for the adsorption of inhibitors on Fe (110) surface (Table 6). The more negative adsorption energy indicates that the system is more stable and the adsorption ability on the metal surface is higher, leading consequently to the best interaction inhibitor molecule/metal surface.^51,52^ All inhibitors exhibit high adsorption energies, on Fe (110) surface increases in the order, PAMP-1 < HAMP-2 < CAMP-4 < HABN-3 which validates the experimental results of inhibition efficiency and the SEM micrographs (see above). As shown in Fig. 9, HABN-3 and CAMP-4 molecules are completely flat and parallel on the Fe (110) surface. This planar conformation leads to a high interaction and large occupation of the metal surface. The results of the molecular dynamic simulations once again confirm those obtained by the electrochemical analyses and surface micrographs. The difference in the adsorption activity between the inhibitors is related to the different substituent groups in the aromatic ring. The use of electron-donating substituents, such as NH, OH and π-electrons in aromatic rings facilitates the transfer of electrons to the vacant Fe-orbital, whereas, the π-antibonding orbitals of the aromatic ring, Cl and CN electron with drawing substituents host electrons from the 4s or 3d orbitals of Fe. In aqueous solution, side views of equilibrium configurations of the four inhibitor molecules are illustrated in Fig. 10. All molecules are also completely flat and parallel on Fe (110) surface. The water molecules can adsorb on the metal surface with higher density than that in the bulk solution. Nevertheless, the close distance between the metal surface and the inhibitor molecules confirm the formation of adhered layer which covers the total metal surface, thus preventing it from aggressive solution attack. The differential energy (d*E*_ads_/d*N*_i_) represents the energy of removing the adsorbent molecule from the metal surface. The differential energy values of the inhibitors (Table 7) are much higher than that of the water molecules, confirming the facility of replacing the water molecules near the metal surface by the inhibitor molecules. Moreover, the adsorption energy of all inhibitors are much higher, resulting in a strong corrosion inhibition efficiency of the organic inhibitors in acid solution.

**Table 6:** Outputs and descriptors calculated by molecular dynamic simulation for the adsorption of inhibitors on Fe (110) surface

Inhibitor	Adsorption energy (kJ mol^−1^)	Rigid adsorption energy (kJ mol^−1^)	Deformation energy (kJ mol^−1^)	d*E*_ads_/d*N*_i_ (kJ mol^−1^)
Fe(110)/PAMP-1	−128.123	−119.307	−8.815	−128.123
Fe(110)/HAMP-2	−135.250	−123.888	−11.361	−135.250
Fe(110)/HABN-3	−141.296	−130.800	−10.495	−141.296
Fe(110)/CAMP-4	−136.254	−129.000	−7.253	−136.254

**Table 7:** Outputs and descriptors calculated from molecular dynamic simulation for the adsorption of inhibitors on Fe (110)/100H_2_O/HCl surface

Inhibitors	Adsorption energy (kJ mol^−1^)	Rigid adsorption energy (kJ mol^−1^)	Deformation energy (kJ mol^−1^)	d*E*_ads_/d*N*_i_, inhibitor	d*E*_ads_/d*N*_i_, H_2_O
Fe(110)/PAMP-1/100H_2_O/HCl	−1501.169	−1559.193	58.024	−111.797	−0.540
Fe(110)/HAMP-2/100H_2_O/HCl	−1421.795	−1476.348	54.552	−133.364	−0.409
Fe(110)/HABN-3/100H_2_O/HCl	−1421.670	−1472.940	51.270	−147.496	−0.582
Fe(110)/CAMP-4/100H_2_O/HCl	−1562.298	−1610.438	48.140	−150.047	−0.810

## 4. Conclusion

New amine derivative compounds were synthesized and successfully used as inhibitors for corrosion of the mild steel in an aggressive environment namely HCl solution. The inhibition performances were related to the substituent group in the phenol ring and the two most efficient inhibitors HABN-3 and CAMP-4. These performances obtained from electrochemical impedance spectroscopy and potentiodynamic polarization were confirmed by surface analysis and a theoretical study. The adsorption of organic molecules on the mild steel surface in HCl solution obeys to Langmuir adsorption isotherm. The molecular dynamic simulations revealed that the four organic molecules were adsorbed practically parallel to the Fe (110) surface and the tendency of the adsorption energies of these molecules increased in the order: PAMP-1 < HAMP-2 < HABN-3 < CAMP-4, which is in total agreement with the experimental results of inhibition activity. This result was confirmed by the quantum chemical calculation which revealed that the inhibitor HABN-3 had the highest tendency to donating electrons to the iron surface.

## Conflicts of interest

There are no conflicts to declare.

## Supplementary Material

RA-010-D0RA03560B-s001
