# Supplementary material for: Experimental and theoretical investigations of four amine derivatives as effective corrosion inhibitors for mild steel in HCl medium
Source: RSC Adv. 2020 Jun 24;10(40):24145–58. doi: 10.1039/d0ra03560b (PMC9055097; doi:10.1039/d0ra03560b)
Supplement: RA-010-D0RA03560B-s001 [file RA-010-D0RA03560B-s001.pdf]

Electronic Supplementary Material (ESI) for RSC Advances.
This journal is © The Royal Society of Chemistry 2020

Experimental and theoretical investigations of four amine derivatives as effective corrosion inhibitors for mild steel in HCl medium

Yasmine Boughoues,[1] Messaoud Benamira,[1,2*] Lyamine Messaadia,[3] Nafila Bouider,[2] Samia Abdelaziz[1]

[1]Laboratory of Interaction Materials and Environment (LIME), University of Mohamed Seddik Benyahia, Jijel, 18000, Algeria

[2]Department of Chemistry, University of Mohamed Seddik Benyahia, Jijel, 18000, Algeria

[3] Laboratoire Énergétique Appliquée et Matériaux, Université de Jijel, 18000, Algeria.

*Corresponding author: m_benamira@univ-jijel.dz; benamira18@yahoo.fr

PAMP-1

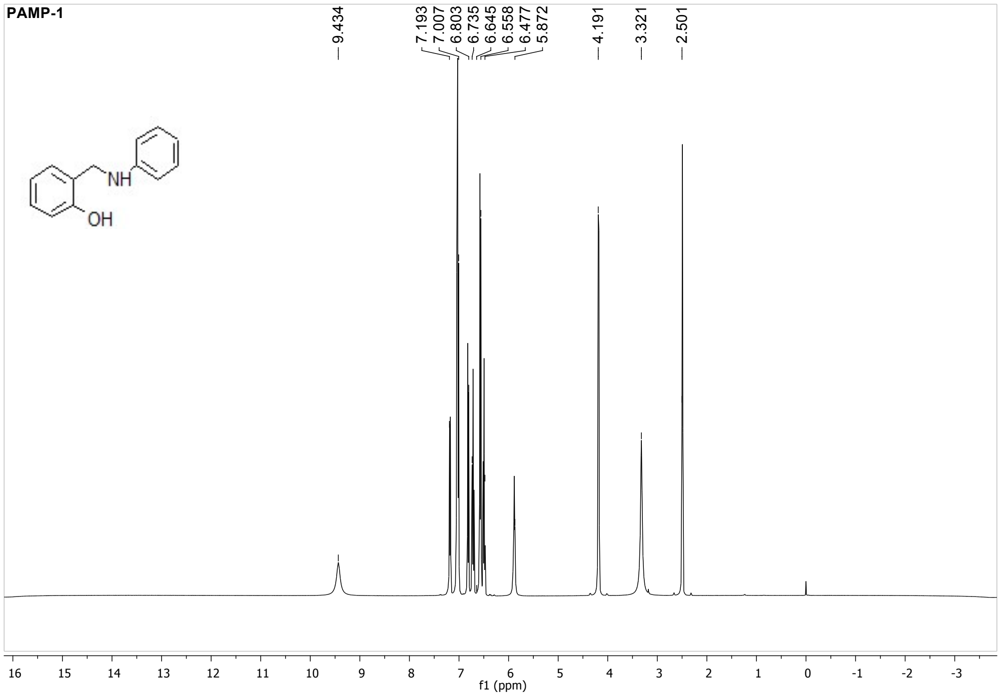
9.434
7.193
7.007
6.803
6.735
6.645
6.558
6.477
5.872
4.191
3.321
2.501
NH
OH
16 15 14 13 12 11 10 9 8 7 6 5 4 3 2 1 0 -1 -2 -3
f1 (ppm)

HAMP-2

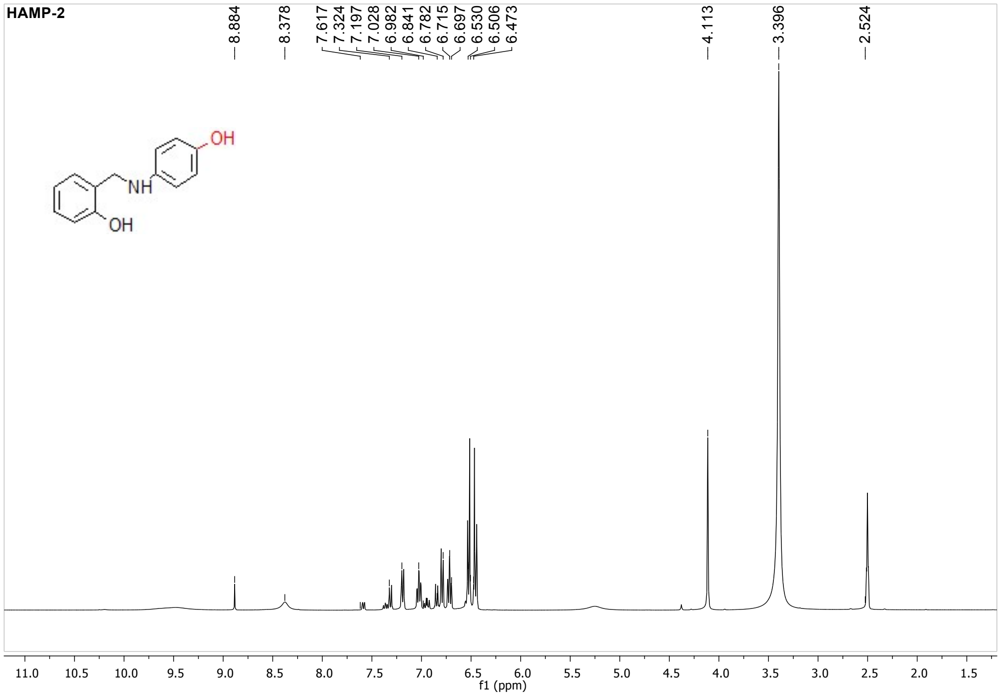
8.884
8.378
7.617
7.324
7.197
7.028
6.982
6.841
6.782
6.715
6.697
6.530
6.506
6.473
4.113
3.396
2.524
OH
NH
OH
11.0 10.5 10.0 9.5 9.0 8.5 8.0 7.5 7.0 6.5 6.0 5.5 5.0 4.5 4.0 3.5 3.0 2.5 2.0 1.5
f1 (ppm)

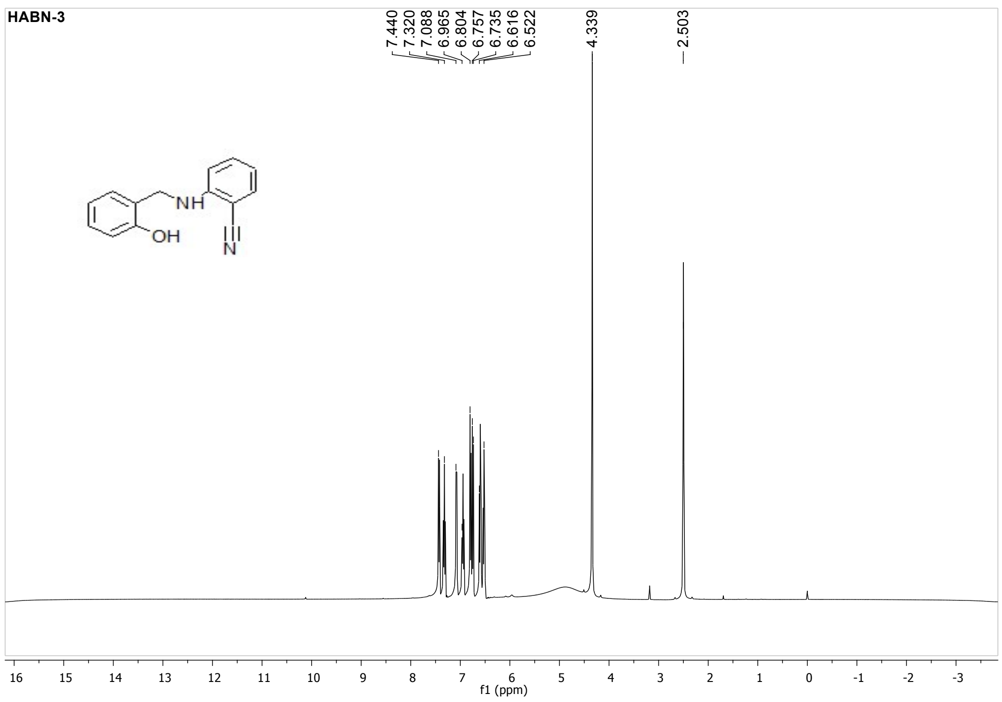

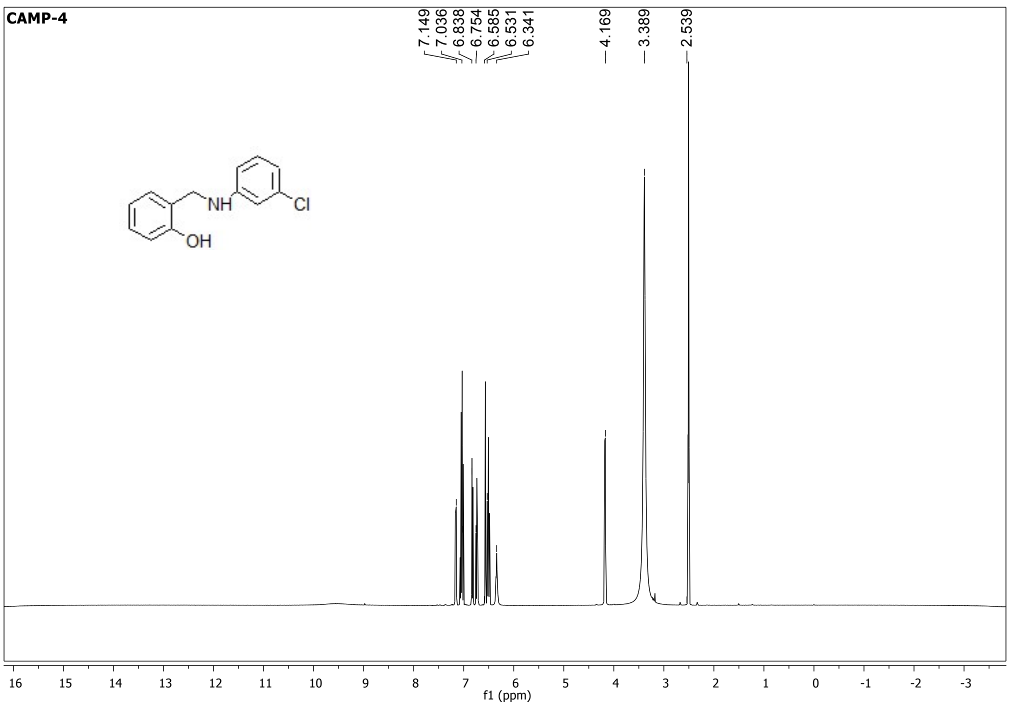

Fig. S1 ^{1}H NHR spectra

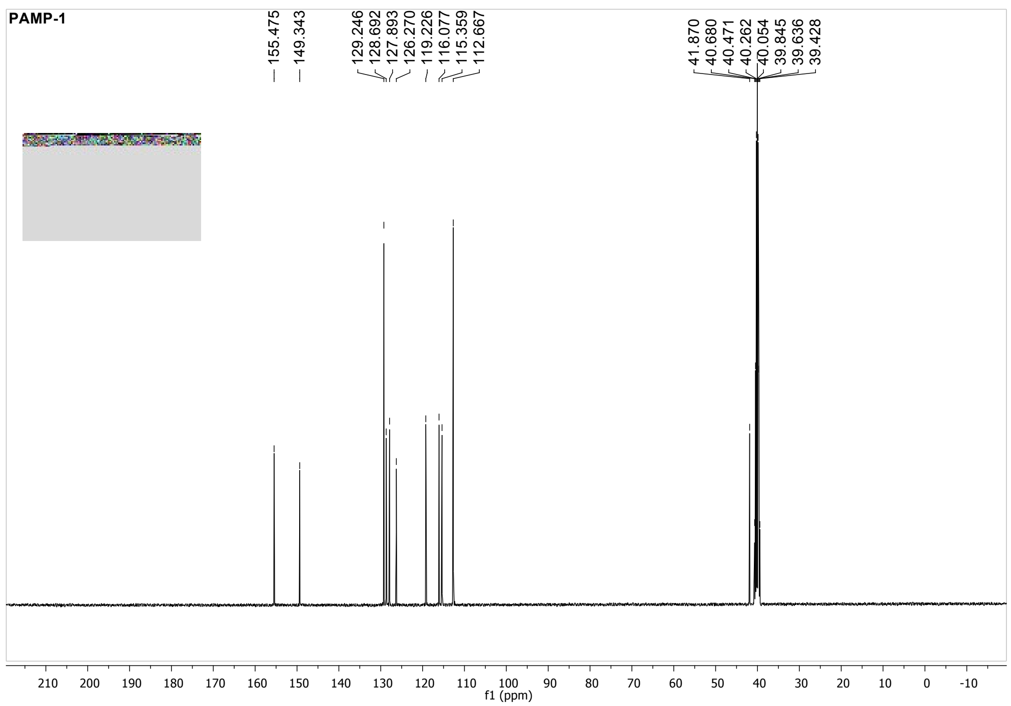
PAMP-1
155.475
149.343
129.246
128.692
127.893
126.270
119.226
116.077
115.359
112.667
41.870
40.680
40.471
40.262
40.054
39.845
39.636
39.428
f1 (ppm)

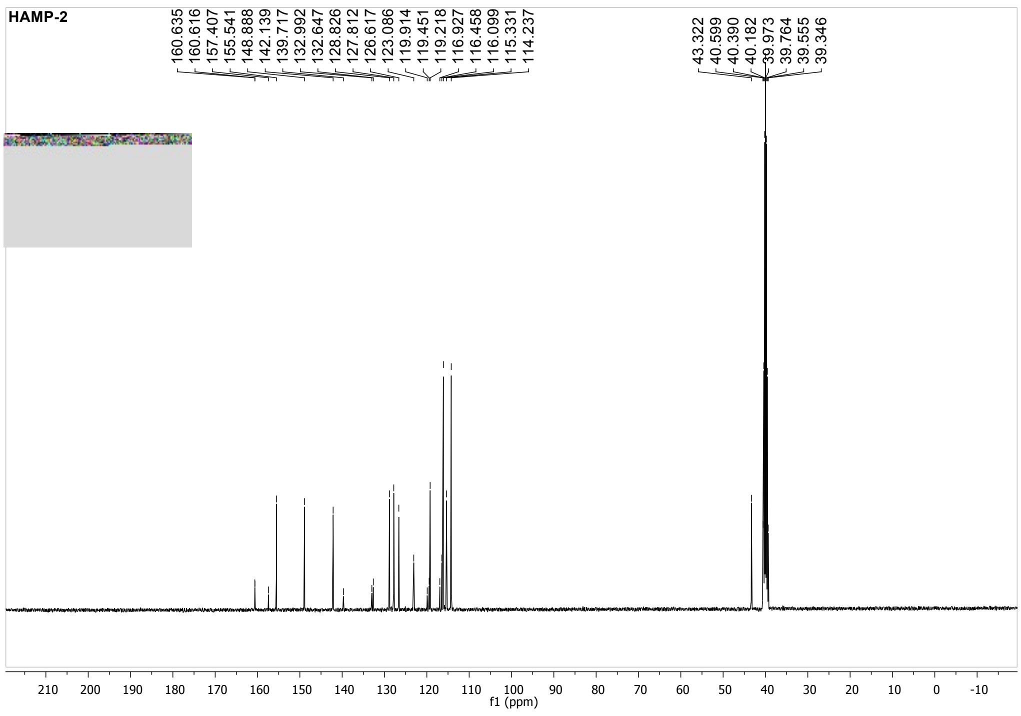
HAMP-2
160.635
160.616
157.407
155.541
148.888
142.139
139.717
132.992
132.647
128.826
127.812
126.617
123.086
119.914
119.451
119.218
116.927
116.458
116.099
115.331
114.237
43.322
40.599
40.390
40.182
39.973
39.764
39.555
39.346
f1 (ppm)

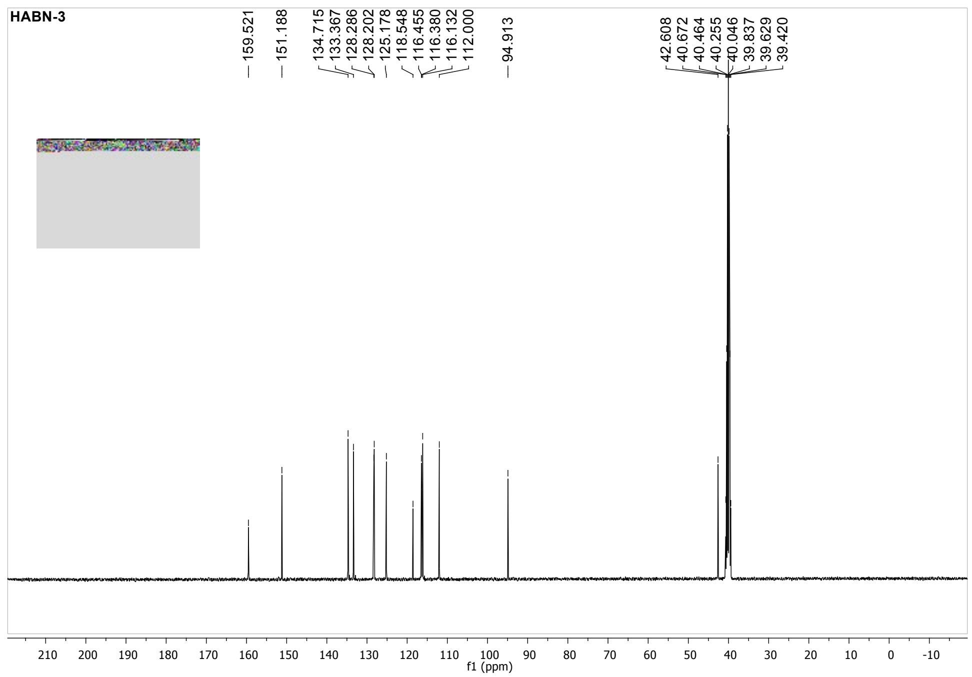

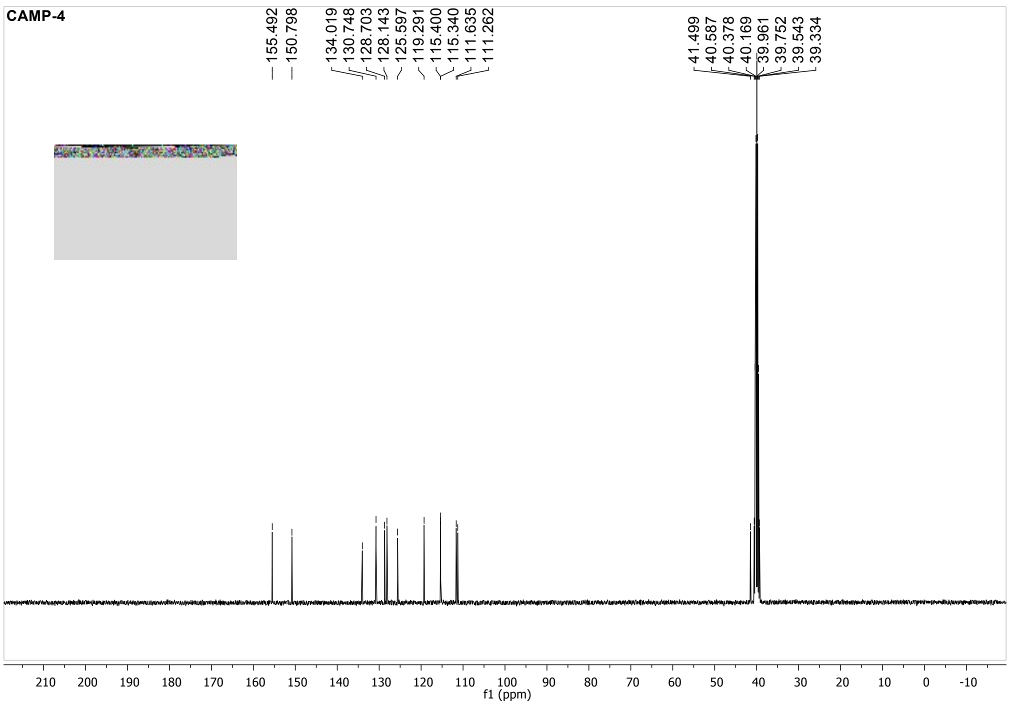

Fig. S2 ^{13}C NMR spectra
